# Artificial Intelligence-Powered Insights into Polyclonality and Tumor Evolution

**DOI:** 10.34133/research.0765

**Published:** 2025-07-02

**Authors:** Hong Zhao, Trey Ideker, Stephen T. C. Wong

**Affiliations:** ^1^ Department of Systems Medicine and Bioengineering, Houston Methodist Neal Cancer Center, Houston Methodist Hospital, Houston, TX 77030, USA.; ^2^ Department of Medicine, Weill Cornell Medicine, New York, NY 10065, USA.; ^3^ Department of Medicine, University of California San Diego, La Jolla, CA 92093, USA.; ^4^ Departments of Radiology, Pathology, and Laboratory Medicine, Weill Cornell Medicine, New York, NY 10065, USA.

## Abstract

Recent studies have revealed that polyclonality—where multiple distinct subclones cooperate during early tumor development—is a critical feature of tumor evolution, as demonstrated by Sadien et al. and Lu et al. in *Nature* (October 2024). These findings show that early polyclonal interactions can overcome fitness barriers, ultimately transitioning to monoclonality as dominant clones emerge. Understanding and targeting these interclonal dynamics offers new therapeutic opportunities. In this perspective, we outline how computational modeling and artificial intelligence (AI) tools can provide deeper insights into tumor polyclonality and identify actionable therapeutic strategies. By applying ligand–receptor interaction analysis, clonal trajectory reconstruction, network and pathway modeling, and spatial analysis, researchers can prioritize communication hubs, evolutionary bottlenecks, and microenvironmental niches that sustain tumor progression. These approaches, when integrated with experimental validation, offer a translational pathway from foundational discoveries to personalized cancer treatments aimed at disrupting cooperative subclonal ecosystems and preventing malignant progression.

We commend the recent *Nature* publications, “Polyclonality overcomes fitness barriers in Apc-driven tumorigenesis” by Sadien et al. [1] and “Polyclonal-to-monoclonal transition in colorectal precancerous evolution” by Lu et al. [2], both featured on 2024 October 30. These groundbreaking studies employed distinct lineage tracing methods to investigate the origins and evolutionary dynamics of colorectal and intestinal tumorigenesis. Despite their different approaches, both studies reached convergent conclusions: Polyclonality plays a pivotal role in the early stages of tumor development, providing critical insights into how diverse cellular populations collaborate to overcome fitness barriers and drive tumor progression.

## Clarifying Definitions: Polyclonality and Monoclonality in Tumor Evolution

In this perspective, we define polyclonality as the presence of multiple independent ancestral lineages contributing to the formation of a single precancerous lesion, each arising separately and harboring distinct sets of somatic mutations. This is consistent with the definition used by Lu et al. [2], who demonstrated through base editor-enabled DNA barcoding that early-stage colorectal lesions often originate from multiple distinct clones. These lineages coexist and interact within the same tissue microenvironment without any single clone initially dominating. The transition to monoclonality refers to the evolutionary bottleneck observed during tumor progression, where competitive pressures—such as selective growth advantages from driver mutations—lead to the outgrowth of one dominant lineage, resulting in a monoclonal tumor mass derived from a single ancestral clone. As shown by Lu et al., this transition is associated with diminished interclonal interactions and a collapse of clonal diversity over time.

## Introduction of the 2 Studies

The Sadien study [1] used multicolor lineage tracing with chemical mutagenesis to demonstrate that many intestinal tumors have a polyclonal origin, consisting of subclones with distinct APC mutations. These diverse subclones are influenced by differences in KRAS and MYC signaling and highlight the role of interclonal interactions in tumor progression. In parallel, Lu et al. [2] employed a base editor-enabled DNA barcoding system to map single-cell phylogenies in mouse models of colorectal tumorigenesis. Their analysis of over 260,000 single cells found that colorectal precancerous lesions often arise from multiple lineages. Early polyclonal lesions displayed extensive intercellular interactions, which were significantly diminished during the transition to monoclonality.

These studies underscore the importance of polyclonality and interclonal interactions in early tumor formation. Intratumor heterogeneity, resulting from clonal evolution and branching, has been widely observed across many cancers, including breast, lung, prostate tumors, and glioblastoma [3,4]. Multiple clones contribute to tumor growth, therapy resistance, and metastasis, emphasizing the need to understand interclonal interactions. Targeting the cooperation or competition between clones also offers opportunities to control tumor progression. Understanding and overcoming these complexities are also crucial in developing effective, long-term cancer therapies for improving cancer treatment outcomes. All of these areas currently rely critically on strong computational analysis tools, and they suggest opportunities for sustained computational method development.

We propose that integrating data from studies like those by Sadien et al. and Lu et al. with advanced computational modeling and artificial intelligence (AI) can yield powerful insights for translational research, from early detection and targeted interventions to personalized treatment plans, ultimately aiming to improve patient outcomes (Figure).

**Figure.**
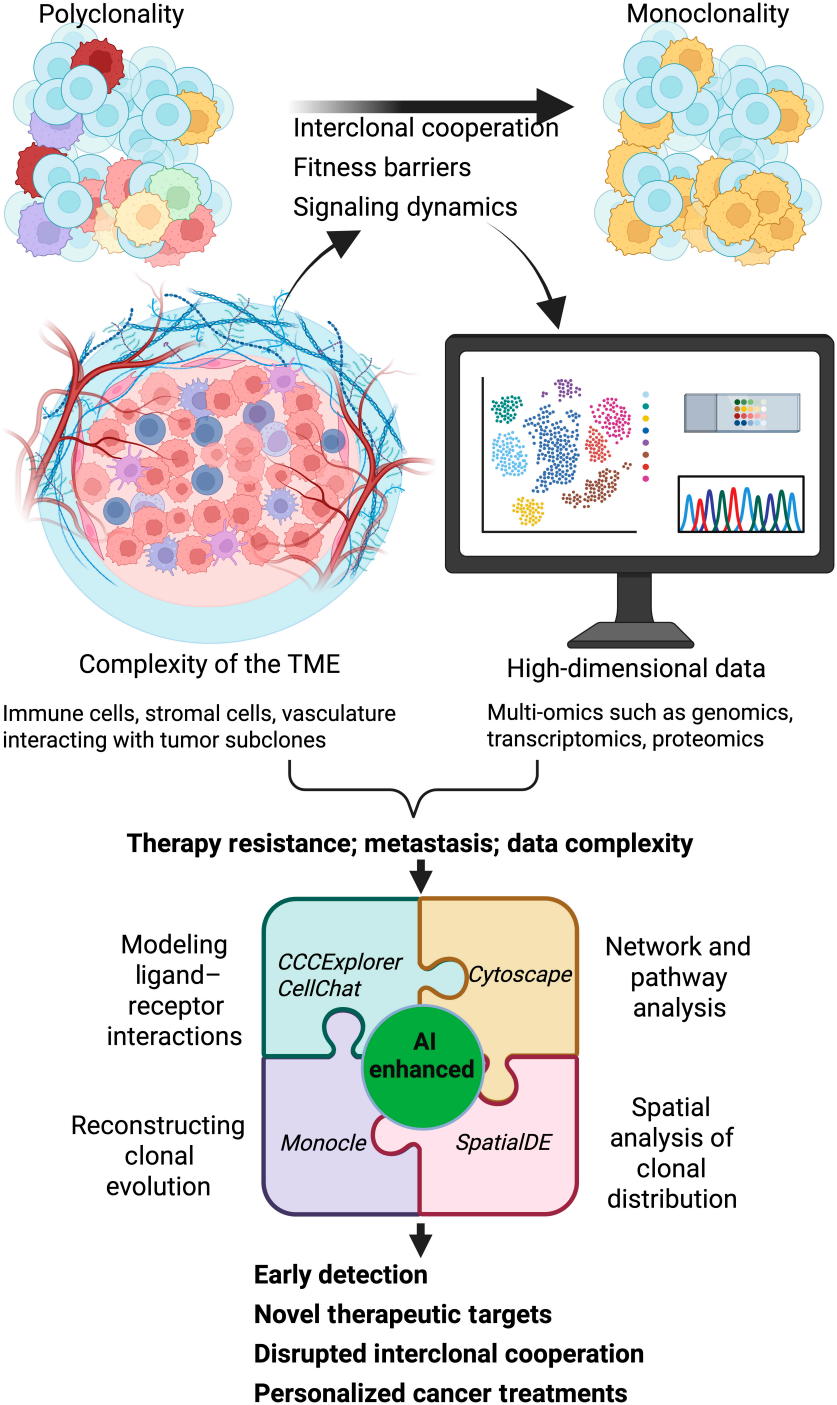
Schematic representation of tumor evolution, interclonal cooperation, and the integration of AI-enhanced tools to address tumor complexity and identify therapeutic targets. The top row illustrates tumor evolution, starting with diverse tumor subclones exhibiting interclonal cooperation, which enables growth and the overcoming of fitness barriers, transitioning toward monoclonality as a dominant subclone emerges. The tumor microenvironment (TME), depicted in the middle left, highlights the complex interplay between tumor cells, immune cells, stromal cells, and vasculature, driving progression and therapy resistance. The bottom right showcases computational and AI tools, including CCCExplorer, CellChat, Monocle, Cytoscape, and SpatialDE, represented as synergistic puzzle pieces used to analyze ligand–receptor interactions, reconstruct clonal trajectories, and explore disrupted signaling networks. Finally, the central integration of these insights directs the identification of novel therapeutic targets and the development of personalized treatment strategies, bridging the gap between foundational research and clinical applications.

### Modeling ligand–receptor interactions

In the context of modeling ligand–receptor interactions, tools such as CellChat [5] can analyze single-cell RNA sequencing (scRNA-seq) data to identify ligand–receptor interactions between subclones. For example, Lu et al. [2] demonstrated 14 enriched ligand–receptor interactions in early polyclonal lesions, involving extracellular matrix (ECM) organization and cell adhesion—processes critical for maintaining clonal cooperation. CCCExplorer [6,7] complements this by mapping how these interactions funnel into downstream signaling pathways. As demonstrated by Lu et al. [2], mutations in genes such as BCL9L, SOX9, TCF7L2, and CTNNB1 diminish interclonal communication during the transition to monoclonality. Tools like CCCExplorer can link these mutations to specific ligand–receptor pathways, revealing how signaling disruptions promote the loss of clonal cooperation.

AI enhances these tools by enabling the prediction and refinement of ligand–receptor interactions. Machine learning algorithms, such as random forests or neural networks, can integrate scRNA-seq data to predict novel ligand–receptor pairs that drive interclonal communication [8]. Autoencoders, a class of unsupervised deep learning models, can be applied to single-cell transcriptomic data to extract subclone-specific latent features. By compressing and reconstructing gene expression profiles, autoencoders can identify signaling modules preferentially activated within distinct subclones. Analysis of these modules can reveal how specific subclones contribute to tumor growth, immune evasion, or therapy resistance [9]. Bayesian network models can be used to infer probabilistic causal relationships between somatic mutations and downstream pathway activation within individual clones. While less established, reinforcement learning could be adapted to simulate clonal competition dynamics and optimize therapeutic strategies that disrupt critical interclonal interactions, using fitness reduction or loss of cooperative signaling as a reward function [10].

Modeling ligand–receptor interactions enables the identification of key interclonal communication pathways that sustain early tumor polyclonality. Therapeutically, targeting these pathways—for example, disrupting ECM remodeling or adhesion signals that maintain clonal cooperation—could selectively destabilize cooperative subclonal ecosystems, impairing tumor progression at its earliest stages.

### Reconstructing clonal evolution with trajectory tools

The reconstruction of clonal evolution, as explored by both Sadien et al. and Lu et al., can also benefit from AI. Tools such as Monocle [11] trace evolutionary trajectories of cell populations, linking specific mutations to changes in clonal behavior. Lu et al. highlighted how mutations in BCL9L and CTNNB1 drive clonal dominance during monoclonal transitions. Deep variational autoencoders (VAEs) can capture branching events in clonal evolution [12], while natural language processing models can annotate mutations like APC or KRAS, contextualizing their functional relevance. Recurrent neural networks and time-series modeling can forecast when subclones gain dominance [13], providing insights into how clonal cooperation diminishes over time.

Reconstructing clonal evolution trajectories allows researchers to map when specific subclones acquire dominance-driving features, such as key mutations or pathway activations. Targeting these critical events therapeutically could delay or prevent the collapse into monoclonality, potentially maintaining a more indolent polyclonal state or rendering tumors more susceptible to therapy.

### Network and pathway analysis

Network and pathway analysis further elucidate how clonal interactions shape tumor evolution. Sadien et al. emphasized diverse signaling profiles among APC-mutant subclones, while Lu et al. linked specific mutations to disrupted ECM and adhesion-related pathways. Tools like Cytoscape [14] can visualize these networks, showing how signaling disruptions lead to clonal competition. Graph neural networks expand on this by identifying cooperative signaling modules [15], while machine learning simulators model how perturbations in nodes like MYC affect interclonal interactions [16]. Deep VAEs, as demonstrated by Geleta et al. [12], can compress complex genotype data into lower-dimensional latent feature spaces while preserving meaningful biological structure. In the context of tumor evolution, similar strategies could be adapted to compress single-cell signaling profiles, where subsequent clustering or anomaly detection analyses might reveal emergent patterns of clonal cooperation or dominance. While promising, these applications remain exploratory and require further validation in cancer models.

Network and pathway analysis can reveal central nodes critical for sustaining interclonal cooperation, such as MYC-driven proliferative signaling or CTNNB1-mediated adhesion. Therapeutic targeting of these central hubs could dismantle the cooperative network architecture, selectively impairing tumor growth and clonal expansion.

### Spatial analysis of clonal distribution

Spatial analysis of clonal distribution, highlighted by Sadien et al. and Lu et al., reveals how subclones occupy distinct niches and interact within the tumor microenvironment. Spatial transcriptomic tools such as SpatialDE [17] map how mutations influence spatial interactions between clones. Lu et al. observed a reduction in interclonal proximity and communication as lesions transitioned to monoclonality. Convolutional neural networks (CNNs) can analyze histological images to track clonal clustering or dispersal [18], while spatial graph convolutional networks quantify cell–cell interactions sustaining polyclonality [19–21]. Agent-based modeling integrates morphological data with AI decision engines, simulating how clones compete or cooperate in real-world conditions [16].

Integrating AI-driven computational analyses with single-cell and spatial omics data offers not only a deeper understanding of early tumor polyclonality but also a practical framework for therapeutic discovery. By identifying interclonal signaling dependencies, reconstructing evolutionary bottlenecks, and mapping cooperative spatial niches [22], these tools can prioritize actionable targets for disrupting subclonal cooperation, delaying clonal dominance, and personalizing treatment strategies for heterogeneous tumors.

Spatial analysis allows mapping of where cooperative or dominant subclones reside within the tumor microenvironment. Therapeutically, spatial targeting—such as site-specific delivery of immune modulators or pathway inhibitors—could be designed to selectively disrupt localized niches supporting polyclonal cooperation or emerging clonal dominance.

### Critical analysis of AI and computational tools

Although computational modeling and AI-driven tools have opened new avenues for studying tumor polyclonality, several technical and practical limitations remain. The predictive power of these models heavily relies on the quality, size, and diversity of input datasets. However, many publicly available scRNA-seq and spatial transcriptomic datasets are relatively small, suffer from sampling biases (e.g., overrepresentation of specific tumor types or stages), and often lack paired clinical annotations such as treatment histories or patient outcomes. These gaps limit the models’ ability to generalize across patient populations.

Furthermore, most AI models, especially deep learning frameworks like CNNs and autoencoders, operate as “black boxes”, producing predictions without clear mechanistic explanations. This lack of interpretability complicates biological validation and limits clinical translation. Overfitting is another major concern, particularly when training datasets are small or unbalanced, leading models to capture noise instead of true biological signals.

Algorithmic biases can also emerge when training data reflect historical inequities or technological artifacts, potentially reinforcing errors rather than correcting them. These issues are especially critical when inferring interclonal communications or reconstructing evolutionary trajectories, where biological nuance matters.

To overcome these challenges, rigorous model validation using independent experimental datasets, cross-cohort comparisons, and standardized benchmarking practices are essential. Expanding multi-institutional data sharing efforts and integrating multi-modal data (e.g., genomics, transcriptomics, proteomics, and imaging) can also improve the robustness and clinical relevance of AI-driven insights into tumor evolution.

## References

[1] Sadien ID, Adler S, Mehmed S, Bailey S, Sawle A, Couturier DL, Eldridge M, Adams DJ, Kemp R, Lourenço FC, et al. Polyclonality overcomes fitness barriers in Apc-driven tumorigenesis. Nature. 2024;634(8036):1196–1203. 39478206 10.1038/s41586-024-08053-0 PMC11525183

[2] Lu Z, Mo S, Xie D, Zhai X, Deng S, Zhou K, Wang K, Kang X, Zhang H, Tong J, et al. Polyclonal-to-monoclonal transition in colorectal precancerous evolution. Nature. 2024;636(8041):233–240. 39478225 10.1038/s41586-024-08133-1

[3] Dentro SC, Leshchiner I, Haase K, Tarabichi M, Wintersinger J, Deshwar AG, Yu K, Rubanova Y, Macintyre G, Demeulemeester J, et al. Characterizing genetic intra-tumor heterogeneity across 2,658 human cancer genomes. Cell. 2021;184(8):2239–2254.e39. 33831375 10.1016/j.cell.2021.03.009 PMC8054914

[4] Gerlinger M, Rowan AJ, Horswell S, Math M, Larkin J, Endesfelder D, Gronroos E, Martinez P, Matthews N, Stewart A, et al. Intratumor heterogeneity and branched evolution revealed by multiregion sequencing. N Engl J Med. 2012;366(10):883–892. 22397650 10.1056/NEJMoa1113205 PMC4878653

[5] Jin S, Guerrero-Juarez CF, Zhang L, Chang I, Ramos R, Kuan CH, Myung P, Plikus MV, Nie Q. Inference and analysis of cell-cell communication using CellChat. Nat Commun. 2021;12(1):1088. 33597522 10.1038/s41467-021-21246-9 PMC7889871

[6] Choi H, Sheng J, Gao D, Li F, Durrans A, Ryu S, Lee SB, Narula N, Rafii S, Elemento O, et al. Transcriptome analysis of individual stromal cell populations identifies stroma-tumor crosstalk in mouse lung cancer model. Cell Rep. 2015;10(7):1187–1201. 25704820 10.1016/j.celrep.2015.01.040

[7] Yeung TL, Sheng J, Leung CS, Li F, Kim J, Ho SY, Matzuk MM, Lu KH, Wong STC, Mok SC. Systematic identification of druggable epithelial-stromal crosstalk signaling networks in ovarian cancer. J Natl Cancer Inst. 2019;111(3):272–282. 29860390 10.1093/jnci/djy097 PMC6410941

[8] Lu M, Sha Y, Silva TC, Colaprico A, Sun X, Ban Y, Wang L, Lehmann BD, Chen XS. LR hunting: A random forest based cell-cell interaction discovery method for single-cell gene expression data. Front Genet. 2021;12: Article 708835. 34497635 10.3389/fgene.2021.708835 PMC8420858

[9] Caravagna G, Heide T, Williams MJ, Zapata L, Nichol D, Chkhaidze K, Cross W, Cresswell GD, Werner B, Acar A, et al. Subclonal reconstruction of tumors by using machine learning and population genetics. Nat Genet. 2020;52(9):898–907. 32879509 10.1038/s41588-020-0675-5 PMC7610388

[10] Sun Z, Chung D, Neelon B, Millar-Wilson A, Ethier SP, Xiao F, Zheng Y, Wallace K, Hardiman G. A Bayesian framework for pathway-guided identification of cancer subgroups by integrating multiple types of genomic data. Stat Med. 2023;42(28):5266–5284. 37715500 10.1002/sim.9911 PMC12167630

[11] Trapnell C, Cacchiarelli D, Grimsby J, Pokharel P, Li S, Morse M, Lennon NJ, Livak KJ, Mikkelsen TS, Rinn JL. The dynamics and regulators of cell fate decisions are revealed by pseudotemporal ordering of single cells. Nat Biotechnol. 2014;32(4):381–386. 24658644 10.1038/nbt.2859 PMC4122333

[12] Geleta M, Montserrat DM, Giro-i-Nieto X, Ioannidis AG. Deep variational autoencoders for population genetics*.* bioRxiv. 2023. 10.1101/2023.09.27.558320

[13] Hewamalage H, Bergmeir C, Bandara K. Recurrent neural networks for time series forecasting: Current status and future directions. Int J Forecast. 2021;37(1):388–427.

[14] Shannon P, Markiel A, Ozier O, Baliga NS, Wang JT, Ramage D, Amin N, Schwikowski B, Ideker T. Cytoscape: A software environment for integrated models of biomolecular interaction networks. Genome Res. 2003;13(11):2498–2504. 14597658 10.1101/gr.1239303 PMC403769

[15] Xingyi Li JX, Li J, Gu J, Shang X. Towards simplified graph neural networks for identifying cancer driver genes in heterophilic networks. Brief Bioinform. 2025;3(26): Article bbae691. 10.1093/bib/bbae691 PMC11697181 39751645

[16] Cogno N, Axenie C, Bauer R, Vavourakis V. Agent-based modeling in cancer biomedicine: Applications and tools for calibration and validation. Cancer Biol Ther. 2024;25(1):2344600. 38678381 10.1080/15384047.2024.2344600 PMC11057625

[17] Svensson V, Teichmann SA, Stegle O. SpatialDE: Identification of spatially variable genes. Nat Methods. 2018;15(5):343–346. 29553579 10.1038/nmeth.4636 PMC6350895

[18] Sharkas M, Attallah O. Color-CADx: A deep learning approach for colorectal cancer classification through triple convolutional neural networks and discrete cosine transform. Sci Rep. 2024;14(1):6914. 38519513 10.1038/s41598-024-56820-w PMC10959971

[19] Li Y, Luo Y. STdGCN: Spatial transcriptomic cell-type deconvolution using graph convolutional networks. Genome Biol. 2024;25(1):206. 39103939 10.1186/s13059-024-03353-0 PMC11302295

[20] Wang H, Zhao J, Nie Q, Zheng C, Sun X. Dissecting spatiotemporal structures in spatial transcriptomics via diffusion-based adversarial learning. Research. 2024;7:0390. 38812530 10.34133/research.0390 PMC11134684

[21] Li Y, Lu Y, Kang C, Li P, Chen L. Revealing tissue heterogeneity and spatial dark genes from spatially resolved transcriptomics by multiview graph networks. Research. 2023;6:0228. 37736108 10.34133/research.0228 PMC10511271

[22] Bhinder B, Gilvary C, Madhukar NS, Elemento O. Artificial intelligence in cancer research and precision medicine. Cancer Discov. 2021;11(4):900–915. 33811123 10.1158/2159-8290.CD-21-0090 PMC8034385

